# Attitudes among paediatric healthcare professionals in Sweden towards sperm donation to single women: a survey study

**DOI:** 10.1186/s40738-020-00078-z

**Published:** 2020-05-07

**Authors:** Gabriela Armuand, Agneta Skoog Svanberg, Claudia Lampic, Evangelia Elenis, Gunilla Sydsjö

**Affiliations:** 1 Department of Clinical and Experimental Medicine, Faculty of Health Sciences, Linköping University, SE-581 83 Linköping, Sweden; 2 Department of Women’s and Children’s Health, Uppsala University, 751 85 Uppsala, Sweden; 3 Department of Women’s and Children’s Health, Karolinska Institutet, 171 77 Stockholm, Sweden; 4 Department of Public Health and Caring Sciences, Uppsala University, 751 22 Uppsala, Sweden; 5 Department of Gynecology and Obstetrics in Linköping, County Council of Östergötland, 581 83 Linköping, Sweden

**Keywords:** Attitudes, Donor assisted conception, Healthcare professionals, Pediatric, Reproductive medicine, Sperm donation

## Abstract

**Background:**

The number of families conceived through sperm donation to single women is increasing. However, there is limited knowledge about health care professionals’ attitudes towards solo-mothers by choice, and there is some indication that professionals’ personal opinions influence their care of individuals who use alternate ways to build a family. The primary aim of the study was to investigate attitudes towards, and experiences of, families following sperm donation to single women among healthcare professionals working in primary child healthcare.

**Methods:**

Between April and November 2016 a total of 712 physicians, registered nurses and psychologists working within primary healthcare in Sweden were invited to participate in a cross-sectional online survey study. The study-specific questionnaire contained the following four domains: Attitudes towards legalization and financing, Attitudes towards the family and the child’s health, Clinical experience and Knowledge about sperm donation to single women.

**Results:**

The majority of the participants were positive or neutral towards sperm donation being allowed to single women in Sweden. However, one third believed that children risk worse mental health and social stigma. Half of healthcare professionals had own clinical experience of caring for solo-mothers by choice and their children, and of these one third perceived that these families had more need of support than other parents. One out of four indicated that they did not have sufficient knowledge to be able to provide adequate care to these families.

**Conclusions:**

The present results indicate that while there was a relatively large support for sperm donation being allowed to single women in Sweden among health care professionals, many expressed concerns about the child’s health, as well as low confidence in their knowledge about the specific needs in this patient group. There is a need for educational interventions targeted to healthcare professionals in primary child healthcare in order to provide adequate care to solo-mothers by choice and their children.

## Introduction

According to Swedish legislation, treatment with gametes from identifiable donors has been permitted for heterosexual couples using donor sperm (1985) or donor oocytes (2003) and for lesbian couples using donor sperm (2005). Sperm donation to single women has only been permitted in Sweden since 2016, but Swedish women have for more than a decade travelled abroad to receive this treatment. As a result, a relatively large number of children to solo-mothers by choice – a concept used both clinically and in research - are already present in the Swedish healthcare system. A Norwegian study showed that solo-mothers by choice were exposed to healthcare professionals’ personal opinions about the mode of conception where they questioned their decision and their competence to care for a child by themselves. As a consequence solo-mothers by choice reported a high threshold for seeking help [1].

Few studies have investigated healthcare professionals’ attitudes towards sperm donation to single women. A Finnish study of medical students showed that over 50% of them were positive towards infertility treatment for single women [2]. Similarly, in a cross-cultural survey study by Záchia et al. (2011) about half of healthcare professionals within the field of assisted reproduction stated that they would perform insemination with donor sperm to a single woman. The decision to perform or not the procedure was found to be influenced by the professionals’ moral values regarding the patient’s right to gestate, the professional’s duty to help the patients, and the child’s right to a father. However, these studies were conducted some years ago and attitudes towards sperm donation to single women may have shifted as a result of societal changes.

By gaining knowledge about healthcare professionals’ perceptions and attitudes regarding sperm donation to single women, we may identify areas in need of interventions in order to optimize the healthcare encounters. The aim of the present study was therefore to investigate attitudes held by Swedish physicians, psychologists, and nurses working in primary child healthcare services towards sperm donation to single women and the implications of this practice. A secondary aim was to investigate whether there was any association between background factors, such as profession, age and sex, and displayed attitudes.

## Materials and methods

### Context

Primary child healthcare services are included in the publicly funded healthcare system in Sweden. The aim of primary child healthcare is to contribute to the best possible physical, mental and social health for children and families [3] and physicians, nurses and psychologists work as a team in order to reach this goal at the primary healthcare centres. Regular well-child visits and vaccinations are provided to all children under the age of six, typically on 12 occasions, of which 10 occur during the child’s first 2 years. These well visits are conducted at the child healthcare centers with a nearly 100% attendance rate. In the present study we refer to single women who have become mothers through sperm donation as solo-mothers by choice.

### Sample and procedures

Participants were recruited from employer mailing lists from 66 primary pediatric healthcare centers, representing four out of 21 counties in Sweden. Information about the study was emailed to a total of 712 physicians, psychologists and registered nurses between April and November 2016. The four counties included both urban and rural areas and covered 14% of Sweden’s total population. The email contained a letter of invitation outlining the study’s objective and methods, as well as a web link to the questionnaire. Three reminders were sent to all possible participants. The study was completely anonymous in order to reduce possible social desirability bias when responding. Completing the questionnaire was regarded as giving informed consent. According to Swedish regulations no ethical approval was needed, as the study did not involve patients and did not measure any medical data or sensitive information.

### Measures

The study-specific questionnaire was developed on the basis of earlier research [4, 5]. It included questions that examined attitudes towards legalization and financing of sperm donation to single women, attitudes towards families created by sperm donation to single women as well as their views on the resulting children’s health, clinical experience of meeting these families, and their knowledge about sperm donation to single women. The survey was evaluated a priori by four individuals representing the target group to make certain that the items were clear and easy to understand. Also, the survey included a short description and an illustration of the process of sperm donation to single women to ensure that the participants had a correct understanding of the procedure. More detailed information regarding the questions posed can be found in the appendices.

An additional five questions measured background variables; age, sex and profession (physician, psychologist or registered nurse), personal experiences of infertility in the individual’s own family or among friends, and whether the individual agreed that a conscience clause (where persons are entitled to refuse to take actions contrary to personal convictions) should be introduced for healthcare professionals in Sweden. The latter item was added because earlier research has shown that religion has an impact on healthcare professionals’ attitudes towards ART (Assisted Reproduction Technology) and other issues related to reproduction [6].

### Data analysis

Comparisons between professional groups were performed with Chi square test (sex and education) and ANOVA (age). Multiple logistic regression models were used to identify factors associated with the attitudes displayed. First, all attitudes (dependent variables) were tested in univariable logistic regression models. The independent variables (age, sex, profession, personal experiences of fertility problems, clinical experience of the patient group, and wanting a conscience clause to be introduced) were chosen based on previous research [4, 6–8]. Entered into multiple logistic regression models were those independent variables that were significantly correlated with any of the displayed attitudes, i.e. sex, age, personal experience of infertility and wanting a conscience clause. The models were evaluated with Nagelkerke’s R2 and percentage of cases were correctly classified. Two models were discarded due to uneven distribution; “It is best for the child if the method of how he/she was conceived is kept secret” and “It is important that the parents are honest with the child with regard to how he/she was conceived”. In order to adjust for multiple comparisons, the Bonferroni correction was used. Comments on what the participants wanted to know more about were analysed by thematic content analysis [9]. Words or phrases reflecting the same content were brought together and formed into categories. Analyses were performed with IBM SPSS statistics 22 (IBM Corp. Westchester County, USA) and significance level was set at *p* < 0.05.

## Results

A total of 208 of the 712 invited healthcare professionals completed the questionnaire (response rate 29.3%), with psychologists having the highest response rate, followed by nurses and physicians (55.9, 35.5 and 17.3% respectively, *p* < 0.001). The mean age was 49.2 years (Standard Deviation [SD] 10.45, range 27–68), with no differences demonstrated between the professional groups or between sexes (men = 48.8 years and wome*n* = 49.2, *p* = 0.878) (Table 1). Half of the participants, 47.6%, had experience of infertility, either personal, in the family or among close friends. One out of five participants wanted a conscience clause for healthcare professionals to be introduced in Sweden (18.3%), with a significant difference noted between the various professional groups (*p* = 0.009).

**Table 1:** Demographics of participants

Characteristics	Registered nurse (*n* = 140)	Physician (*n* = 49)	Psychologist (*n* = 19)	*P*^a^
Age (mean, SD)	49.8 (10.32)	48.5 (10.30)	46.8 (11.78)	NS
	n (%)	n (%)	n (%)	
Sex ^b^				< 0.001
Female	139 (99.3)	32 (65.3)	18 (94.7)	
Male	0	17 (34.7)	1 (5.3)	
Own experience of infertility				0.048
Yes	59 (42.1)	27 (55.1)	13 (68.4)	
No	81 (57.9)	22 (44.9)	6 (31.6)	
Wanting a conscience clause ^b, c^				0.009
Yes	23 (16.4)	15 (30.6)	0	
No	115 (82.1)	34 (69.4)	19 (100)	

^a^ Between professional groups; ^b^ Percentages do not sum to total due to missing values; ^c^ Conscience clause = where persons are entitled to refuse to take actions contrary to personal convictions;

### Attitudes towards legalization and financing

The majority, 81.7%, of the participants were positive or neutral towards legalization of sperm donation to single women in Sweden (Table 2), while a smaller percentage were positive or neutral towards it being publicly funded (57.7%). Multivariable regression models revealed that wanting a conscience clause to be introduced in Sweden was strongly associated with a negative attitude towards legalizing sperm donation to single women (Odds Ratio [OR] 5.09, 95% Confidence Interval [CI] 2.05–12.67) or publicly financing it (OR 2.81, 95% CI 1.22–6.43). Sex, age and having a personal experience of infertility had no independent impact on displayed attitudes.

**Table 2:** Proportion of the professionals who agreed with, or were neutral about, the legalization and financing of sperm donation to single women ^a^

Attitudes ^b^	Total	Registered nurse	Physician	Psychologist
	n (%)	n (%)	n (%)	n (%)
Sperm donation to single women should be allowed	170 (81.7)	118 (84.3)	35 (71.4)	17 (89.5)
Sperm donation to single women should be publicly funded	120 (57.7)	87 (62.1)	22 (44.9)	11 (57.9)
The child should have the right to know the sperm donor’s ID	172 (87.3)	112 (84.8)	42 (91.3)	18 (94.7)

^a^ All participants did not answer all questions; ^b^ Indicating 3 to 5 on a five-point Likert scale (Strongly agree/Agree/Neutral)

### Attitudes towards the family and the child’s health

The responses regarding the attitudes towards the family and the child’s health are presented in detail in Table 3. The vast majority considered the single mother as being capable of caring for her child (89%). A lower percentage, albeit still the majority of participants, felt that two parents are essential (64%). Multivariable regression models showed that wanting a conscience clause was more often associated with agreeing that a child needs two parents (OR 3.97, 95% CI 1.40–11.27), disagreeing with the statement saying that children being conceived through sperm donation to single women are as healthy as other children (OR 2.26, 95% CI 1.01–5.08), and believing that the child risks social stigmatization (OR 3.80, 95% CI 1.66–8.70). Also, those who wanted a conscience clause more often believed that the relationship between child and parent could be damaged if the child learns about the mode of conception (OR 3.61, 95% CI 1.25–10.42). Younger participants more often agreed that it would be good for the child to be able to know the identity of the donor (OR 1.04, 95% CI 1.01–1.08), as did women compared to men (OR 3.31, 95% CI 1.07–10.29). Women also more often agreed with the statement saying that single mothers can very well take care of a child on their own (OR 6.07, 95% CI 1.59–23.19). Having one’s own experience of infertility had no independent impact on the attitudes displayed.

**Table 3:** Proportion of healthcare professionals agreeing ^a^ with statements about solo-mother by choice families

Attitudes	Total ^b^	Registered nurse	Physician	Psychologist
		n (%)	n (%)	n (%)
A child needs two parents	127 (64.1)	89 (66.4)	30 (63.8)	8 (47.1)
Single mothers can very well take care of a child on their own	176 (89.3)	122 (91.7)	36 (80.0)	18 (94.7)
Single women with children conceived through sperm donation shall have the same right to child support as other single parents	131 (65.5)	88 (65.7)	29 (61.7)	14 (73.7)
The child is as healthy as other children	119 (60.1)	81 (60.9)	27 (57.4)	11 (61.1)
The child risks worse physical health	39 (19.9)	27 (20.5)	9 (20.0)	3 (15.8)
The child risks worse mental health	64 (32.2)	43 (32.3)	16 (34.0)	5 (26.3)
Single women with children conceived through sperm donation are more involved in their children compared to mothers in other families	49 (25.0)	38 (28.8)	8 (17.8)	3 (15.8)
The child may experience a social stigma	75 (37.7)	47 (35.3)	19 (40.4)	9 (47.4)
When mature enough, it is good for the child to be able to know the identity of the donor	139 (70.2)	91 (68.9)	32 (68.1)	16 (84.2)
It is best for the child if the method of how he/she was conceived is kept secret throughout life	4 (2.0)	4 (3.0)	0	0
It is important that the parents are honest with the child with regard to how he/she was conceived	185 (93.4)	123 (93.2)	43 (91.5)	19 (100)
The child’s relationship with the parent could be damaged if he/she learns about the mode of conception	24 (12.2)	17 (12.9)	6 (13.0)	1 (5.3)
Contact with the donor (when mature enough) can be harmful for the offspring and/or the family	12 (6.0)	10 (7.5)	2 (4.3)	0

^a^ Indicating 4 or 5 on a five-point Likert scale (Strongly agree/ Agree); ^b^ All participants did not answer all questions;

### Clinical experiences and perceived need for more knowledge

About half of participants (53%), had clinical experience with families who had children through sperm donation to single women, with no significant difference between professional groups (80 nurses, 20 physicians and 11 psychologists, *p* = 0.143). Of these, 36 (32.7%) perceived that these families had more need of support than other parents, and six had referred the child and/or the family to specialist care. These included three referrals to a psychologist at the children’s hospital, two referrals to a counsellor, and one referral to a dietician.

In the total group of participants, one in four (27.4%) indicated that they did not have sufficient knowledge to be able to provide adequate care for families conceived through sperm donation to single women, with no difference noted between the professional groups. However, those who had no prior clinical experience more often felt that they had limited knowledge compared with those who had some kind of experience with the patient group (no clinical experience = 38.8% vs clinical experience = 22.2%, *p* = 0.011). About half of the total group of participants (52.4%) indicated that they wanted to know more about sperm donation to single women. Thematic content analysis of written responses provided by 88 participants (13 physicians, 65 nurses, 10 psychologists) identified four areas in need of improved knowledge, each identified in italics in the following section.

In “*Knowledge about the process of sperm donation to single women*” the participants called for information about how the treatment was performed, and how the sperm donors and the single women applying for sperm donation were evaluated. In “*Knowledge about psychosocial and psychological aspects of sperm donation to single women*” the participants stated that they wanted to know more about the family’s psychological wellbeing, especially from the perspective of the child, including how the child dealt with the disclosure of the sperm donation and being brought up by only one parent. In “*Knowledge about laws and regulations*” the participants wanted to know more about the legal aspects of sperm donation to single women, both regarding legal rights and obligations for donors and recipients, as well as the child’s rights. In the area “*Knowledge about the healthcare professionals’ role and how to support the single mother”* the participants called for information to clarify their role as healthcare professionals within paediatric healthcare, how they could best support single women in their role as parents, and about how to disclose the sperm donation to the child.

## Discussion

The study results showed that the majority of professionals working within primary child healthcare in Sweden were positive or neutral towards sperm donation being allowed for single women, whilst fewer agreed that it should be publicly funded. Even though more than half of the participants agreed that a child needs two parents, the majority thought that a single mother could very well take care of a child on her own. However, one out of three believed that children to solo-mothers by choice risk worse mental health and social stigma, even though that notion is not scientifically substantiated.

The majority (82%) of the participants working in primary child healthcare were positive or neutral towards sperm donation being allowed in Sweden, a finding comparable with results from a previous study among the Swedish general population (76%) [10]. The proportions of healthcare professionals’ attitudes towards legalization of the different modes of conception mirror very well the ongoing discussions and changes in Sweden and likely in other countries as well; legislation allowing sperm donation to single women in Sweden was passed in 2016 shortly after the start of our study. In the study by Záchia et al., (2011), including professionals from countries with different legislation regarding donor insemination for single women, about half stated that they would perform the procedure and legal aspects did not appear to be insurmountable obstacles for their decision. Instead, moral values regarding reproductive rights, duty to help the patient and the child’s right to a father were found to be of importance.

Research about the health of children conceived through sperm donation to single mothers is limited and based on small samples, but data shows socio-emotional development [11, 12] and child adjustment [13] within a normal range. Despite these reassuring findings, our study found that one third of the healthcare professionals believed that the children risked having worse mental health. However, the healthcare professionals were aware of their lack of knowledge and wanted to know more about the family’s psychological wellbeing. That urge is further corroborated by the findings of Zadeh et al. [14] demonstrating that solo mothers by choice often receive external criticism by their social network regarding their untraditional choice of parenthood, which in turn emphasizes the need for counselling and psychological support to these families, both prior to, as well as after childbirth [14].

In 1985 Sweden became the first country to implement mandatory gamete donor identification legislation on the basis of the donor offspring’s right to receive information on his/her genetic origin. The present results showed that the majority of the healthcare professionals thought that the parent should be honest with the children about the mode of conception and that it would be beneficial for the child to be able to know the identity of the donor. Previous research showed that a higher proportion of solo-mothers by choice intended to disclose to their children the use of sperm donation compared to partnered mothers, which was ultimately attributed to the absence of a father [15]. However, they might sometimes be unwilling to disclose this piece of information to their surrounding social network and instead use self-shielding strategies, encouraged also by staff at the fertility clinic [14]. Research has shown that the starting point of talking about the sperm donation treatment often was catalysed by the child’s questions about why he/she did not have a father as other children have [16]. Published children’s books targeting the lay audience and aiming to explain to children about conception by gamete donation (eggs or sperm) are available in several languages, including English and in Swedish. However, a study of donor-conceived children found that these children wished that their parents had received counselling about how to talk with the child about donor conception at several stages during the child’s life [17]. It is reasonable to believe that many solo-mothers by choice will turn to a primary healthcare provider for advice regarding disclosure issues. Our finding that half of the healthcare professionals wanted to know more about sperm donation to single women, including how they could support these women in their communication with their child about the donor-conception, indicates a need for educational resources directed at professionals in primary child healthcare. So far, the recommendations available have mostly focused on fertility specialists or donor recipients. The Ethics Committee of the American Society of Reproductive Medicine has issued an official opinion on gamete donation discussing the ethical implications for the involved parties while encouraging disclosure [18].

A strength of the present study is that we invited all healthcare professionals working in primary child healthcare in four counties in Sweden to participate, physicians, nurses and psychologists alike. However, as the study was performed between April and November 2016, it is possible that some individuals on the mailing lists were not clinically active during the data collection period and therefore did not receive the study invitation. The anonymous study design reduced the risk of responses being influenced by social desirability, but it also made comparisons between responders and non-responders impossible. It is also important to keep in mind that there is a risk of selection bias related to the topic of the study. However, it is not known if this would entail that those supporting or opposing the introduction of new ARTs, such as sperm donation to single women, might have participated to a higher extent. The low response rate (29.3%) limits the generalizability of our findings and the results must be therefore interpreted with caution. The higher response rate among psychologists (55.9%) may be due to the psychological aspects of sperm donation being more interesting for this group compared to physicians and registered nurses.

## Conclusions

Keeping the low response rate in mind, the majority of the healthcare professionals working in primary child healthcare were positive or neutral towards sperm donation being allowed to single women in Sweden. However, one fourth indicated not having sufficient knowledge about this patient group’s specific needs to provide adequate care. Targeted educational interventions may increase child health care professionals’ ability to support solo-mothers by choice in their communication with the child about the donor-conception.

## Data Availability

The datasets used in the present study are available from the corresponding author on reasonable request.

## Appendix

The study-specific questionnaire was developed on the basis of earlier research, theory and clinical experience. Previously used items to measure attitudes among healthcare professionals within reproductive medicine were adapted and used in the present study [4, 5]. In order to ensure that participants had a correct understanding of sperm donation to single women, the questionnaire contained an illustration showing the steps involved in the process. The questionnaires’ feasibility and face validity were evaluated by professionals in the target group, and minor changes were made.

Attitudes towards legalization and financing were measured by three statements: that sperm donation should be allowed to single women, that it should be publicly funded and that the child should have the right to know the sperm donor’s ID (5-point Likert scale ranging from “strongly disagree” to “strongly agree”, dichotomized into “negative attitude” and “neutral/positive attitude”).

Attitudes towards families by solo-mothers by choice and their children’s health were measured by 14 statements, e.g. “Children to solo-mothers by choice risk having worse physical health” (5-point Likert scale ranging from “strongly disagree” to “strongly agree”, dichotomized into “disagree/neutral” and “agree”).

Clinical experience of meeting solo-mothers by choice families was assessed by three items: if they, in their clinical work, had met families following sperm donation to single women (yes/no), and if they had done so, how they perceived these single parents’ need for support in comparison to other parents’ needs (5-point Likert scale ranging from “much lower” to “much higher”). Those who reported “somewhat higher” and “much higher” were requested to state whether they had referred any of these families to specialist care for problems associated with the mode of conception, and in that case, where they had referred them.

Knowledge about sperm donation to single women was measured by two items: if there was something about the mode of conception that they wanted to know more about (yes/no), and in that case, what they wanted to know more about (open-ended format). Confidence in knowledge was assessed with one statement, “I feel that I have sufficient knowledge about sperm donation to single women and what it may imply for the child and family in order to provide adequate care” (5-point Likert scale ranging from “strongly agree” to “strongly disagree”, dichotomized into “Agree/Neither nor” and “Disagree”).

## Abbreviations

ART: Assisted reproduction technology
CI: Confidence interval
OR: Odds ratio
SD: Standard deviation
